# Pre-Competition Anxiety and Mood State in White-Belt Brazilian Jiu-Jitsu Athletes: An Exploratory Comparison Between Medalists and Non-Medalists

**DOI:** 10.3390/sports14060216

**Published:** 2026-05-26

**Authors:** Marcelo Victor Menezes Santana, Felipe J. Aidar, Renato Méndez-DelCanto, Marcio Getirana-Mota, Alfonso López Díaz de Durana, Rapahel Fabricio de Souza, Ciro José Brito, Teresa Figueiredo, Luis Leitão

**Affiliations:** 1 Graduate Program in Movement Science, Federal University of Sergipe (UFS), São Cristovão 49100-000, SE, Brazil; marcelovictorms@gmail.com (M.V.M.S.); fjaidar@gmail.com (F.J.A.); renato.mendez@usach.cl (R.M.-D.); marcio_getirana@hotmail.com (M.G.-M.); raphaelfs@academico.ufs.br (R.F.d.S.); 2 Department of Physical Education, Federal University of Sergipe (UFS), São Cristovão 49100-000, SE, Brazil; 3 Group of Studies and Research of Performance, Sport, Health and Paralympic Sports—GEPEPS, Federal University of Sergipe (UFS), São Cristovão 49100-000, SE, Brazil; 4 Graduate Program of Physiological Science, Federal University of Sergipe (UFS), São Cristovão 49100-000, SE, Brazil; 5 Facultad de Ciencias de al Actividad Fisica y del Deporte (INEF), Universidad Politécnica de Madrid, 28040 Madrid, Spain; alfonso.lopez@upm.es; 6 Graduate Program of Physical Education, Federal University of Juiz de Fora, Juiz de Fora 36036-900, MG, Brazil; cirojbrito@gmail.com; 7 Instituto Politécnico de Setúbal, Escola Superior de Educação, CIEQV, 2910-761 Setúbal, Portugal; teresa.figueiredo@ese.ips.pt; 8 Life Quality Research Centre (CIEQV), 2910-761 Setúbal, Portugal; 9 Research Centre in Sports, Health and Human Development (CIDESD), 6201-001 Covilhã, Portugal

**Keywords:** combat sports, psychological state, amateur athletes, sport performance

## Abstract

Background: Combat sports performance is highly determined by psychological factors, and differences in pre-competitive anxiety and mood states can exist between medalist and non-medalists athletes; Methods: The present study aims to assess pre-competitive anxiety and mood state differences between medalist and non-medalists Brazilian Jiu-Jitsu (BJJ) athletes graded as white-belts. The Competitive State Anxiety Inventory–2 (CSAI-2) and the Brunel Mood Scale (BRUMS) questionnaires were applied to 26 BJJ white-belt athletes before a fight in a national-level competition.; Results: Medalists presented less cognitive anxiety (22.36 ± 3.82 vs. 25.21 ± 3.17; *p* < 0.05) and higher mental confusion (9.86 ± 3.01 vs. 7.43 ± 3.01; *p* < 0.05) than non-medalist athletes. No significant differences were found in any other variable; Conclusions: The relationship between pre-competitive anxiety and sport performance is clear; however, higher mental confusion in medalists is a confounding result. More research is needed on this topic to elucidate the psychological phenomena of higher mental confusion in less-experienced athletes.

## 1. Introduction

Success in competitive sport is a complex phenomenon believed to depend mostly on physical, technical, and tactical aspects. However, psychological factors can be crucial to winning a competition [1]. Among these factors, anxiety is highlighted for impairing sports performance, as it usually represents a negative psychological state characterized by heightened mental tension, excessive worry, and feelings of apprehension [2]. During sports practice, competitions can be an anxiogenic stimulus that can trigger the so-called pre-competitive anxiety. In this context, athletes can suffer physiological, motor, and cognitive alterations that could affect physical, technical, and tactical performance during the competition [3]. Another important psychological factor for competition performance is an athlete’s mood state before the contest. Mood state can be used to predict the athletes’ success in a competition event, and some aspects that compound it, such as depression, confusion, and vigor, can directly impact the sport performance [4].

Pre-competitive anxiety and mood states can be measured through a variety of tools in the sport context. The Competitive State Anxiety Inventory–2 (CSAI-2) is a questionnaire widely used in sports that measures somatic anxiety, cognitive anxiety, and the athletes’ self-confidence [5]; while the Brunel Mood Scale (BRUMS) is a mood profiling scale that can predict sport performance through screening the athletes mood state [6]. As an important relationship between these psychological factors and sport performance exists, medalist and non-medalists athletes could present differences in these two variables. This has been demonstrated in combat sport athletes participating in boxing [7], judo, and Brazilian Jiu-Jitsu [8].

Brazilian Jiu-Jitsu (BJJ) is a grappling combat sport where athletes force opponents into submission, performing intermittent high-intensity efforts that can produce significant amounts of fatigue [9]. For its exhausting nature, a BJJ competition can be perceived by athletes as a hard challenge [10]. In addition, combat sports such as BJJ are known for demanding high mental toughness due to frequent exposure to harmful stimuli and injury risks during competition. This can trigger anxiety symptoms like anticipatory fear and apprehension, which could affect the fight’s success [11]. In a study from Andreato et al. [12], blue belt BJJ athletes perceived the fights as hard or very hard, and reported moderate anxiety levels and a vigorous mood state before the fight. However, this study does not examine differences between medalists and non-medalists.

Until now, research on psychological factors and winning and losing outcomes has been conducted on experienced BJJ athletes. However, experienced athletes can suffer fewer negative psychological symptoms than novice athletes [13], and we still do not know how pre-competitive anxiety can affect novice BJJ fighters and which type of mood state can predominate in this population. Therefore, this study aims to investigate the differences in pre-competitive anxiety and mood state between medalist and non-medalist BJJ athletes graded as white belts.

## 2. Materials and Methods

### 2.1. Sample

This is a cross-sectional observational study with a sample of 28 Brazilian Jiu-Jitsu male athletes. Table 1 shows the characterization of the sample. The sample size was calculated a priori using the “F (ANOVA)” statistic in the G*Power^®^ software (Universidade Heinrich Heine de Düsseldorf, Version 3.0; Düsseldorf, Germany), considering an α < 0.05 and a β = 0.80. Thus, we considered the effect of ƒ2 0.121 for pre-competitive anxiety in fighters found in another study. Therefore, an effect size = 0.371 was generated to calculate the sample size, through which a minimum sample size of 26 subjects was defined, with an estimated power of 0.80 [7].

Athletes who finished in the top three were considered medalists, and athletes who did not finish in the top three of their categories were considered non-medalists. Only athletes who voluntarily participated in the study are included.

In Brazilian Jiu-Jitsu (CBJJ), weight categories are divided by belt and weight (with kimono), ranging from Roosterweight to Super Heavyweight. In the adult male category, as a sample for this study, the weight categories are: Roosterweight (57.5 kg), Featherweight (64 kg), Lightweight (70 kg), Middleweight (76 kg), Light Heavyweight (82.3 kg), Light Heavyweight (88.3 kg), Heavyweight (94.3 kg), Super Heavyweight (100.5 kg), and Super Heavyweight (no limit). Thus, in the Featherweight category, we had three medalists (M) and four non-medalists (NM), in the Lightweight category, we had four athletes in each category, in the Lightweight category, we had four medalists and three non-medalists, and in the Middleweight category, we had four medalists and two non-medalists. We had 15 medalist athletes (54%) and 13 non-medalist athletes (46%). Among the medalists, we had Gold Medal winners (*n* = 4, 27%), Silver Medal winners (*n* = 4, 27%), and Bronze Medal winners (*n* = 7, 47%).

Eligibility criteria included (1) being an active competitor and (2) having their match confirmed following the official weigh-in. Athletes who did not complete the questionnaires correctly or whose matches were canceled at the last minute were excluded from the study. All athletes signed an informed consent form following the 466/2012 resolution of the National Commission for Research Ethics of the Brazilian National Health Council. The study was also approved by the Ethics Committee of the Federal University of Sergipe (UFS) by protocol number (ID-CAAE: 67953622.7.0000.5546) under technical report 6,523,247, dated 22 November 2023. All procedures were executed according to the 2013 Helsinki Declaration from the World Medical Association.

### 2.2. Instruments and Procedures

**Figure 1:**
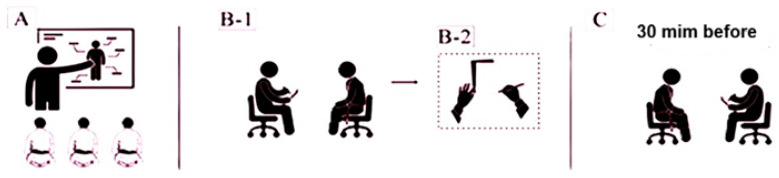
Procedures adopted for data collection. (**A**) Sample recruitment and explanations of the study’s objectives, risks and benefits. (**B-1**) Initial interview in which data were collected on the respective categories of the participants, and, later, the questionnaires for the analysis of anxiety were explained. (**B-2**) Signing the Free and Informed Consent Term (FICT). (**C**) Application of the questionnaires (30 min before the competitions of the respective categories).

Both mood state and pre-competitive anxiety were evaluated through questionnaire application during the interval between the athletes’ weighing and the start of the respective fight of each athlete. Questionnaires were taken approximately 30 min before the start of the fights. Later, 30 min before the start of the competitions of their respective categories, the athletes were individually interviewed by a psychologist, who applied and collected the answers to the anxiety questionnaires (Figure 1). At the end of the competition, the respective placements of the athletes who participated in this study were recorded. Subsequently, for data analysis, the sample was divided into groups: Gold-Medal (25%), Silver-Medal (30%), Bronze-Medal (22%) and the Control Group, formed by non-medalist athletes (27%).

Mood states were assessed using the Brunel Mood Scale (BRUMS) questionnaire. This is a 24-item scale including 6 subscales, each corresponding to a mood dimension: tension, depression, anger, vigor, fatigue, and mental confusion [14]. Each item can be rated from 0 (“none”) to 4 (“extreme”), and each dimension score ranges from 0 to 16. In addition, BRUMS has been correctly translated to Portuguese and validated in the Brazilian population [15], and has been widely used to asses athletes mood state in sport contexts [6].

Pre-competitive anxiety was assessed using the Competitive State Anxiety Inventory–2 (CSAI-2) questionnaire that was validated in the Brazilian population by Bartholomeu et al. [16]. This 27-item scale includes 3 subscales: cognitive anxiety, somatic anxiety, and self-confidence, with a response scale ranging from 1 to 4 (1 = nothing, 2 = something, 3 = moderate, and 4 = great) [17]. Following the Alejo et al. [7] methods, A Brazilian Portuguese translation adapted version of the CSAI-2 [5,17], was used. This instrument consists of 27 items, grouped into three factors, as follows: items 1, 4, 7, 10, 13, 16, 19, 22, and 25 belong to the cognitive anxiety factor; 2, 5, 8, 11, 14 (inverted item), 17, 20, 23, and 26 to somatic anxiety; and 3, 6, 9, 12, 15, 18, 21, 24, and 27 to self-confidence. Finally, the sub-scale scores were interpreted as low (9 to 18 points), average (19 to 27 points), or high (28 to 36 points).

### 2.3. Statistical Analysis

Descriptive statistics was performed using the measures of central tendency, mean (X) ± standard deviation (SD), and 95% confidence interval (95% CI). To verify the normality of the variables, the Shapiro–Wilk test was used in view of the sample size. The data for all variables analyzed were homogeneous and normally distributed. If normality was observed, an independent *t*-test was performed to determine the differences between medalists and non-medalists. If the normality assumptions were not met, the Mann–Whitney test was used. A statistical significance level of *p* ≤ 0.05 was adopted. The effect size was calculated using Cohen’s “d”. A “d” value < 0.2 was considered a trivial effect, 0.2 to 0.6 a small effect, 0.6 to 1.2 a moderate effect, 1.2 to 2.0 a large effect, 2.0 to 4.0 a very large effect, and ≥4.0 an extremely large effect [18]. Cohen’s “d” was calculated as the difference between the mean divided by the pooled SD to estimate the effect size for between-lift comparison [19]. Statistical analyses were made with the Statistical Package for the Social Sciences (SPSS) version 25.0 (IBM, New York, NY, USA).

## 3. Results

**Figure 2:**
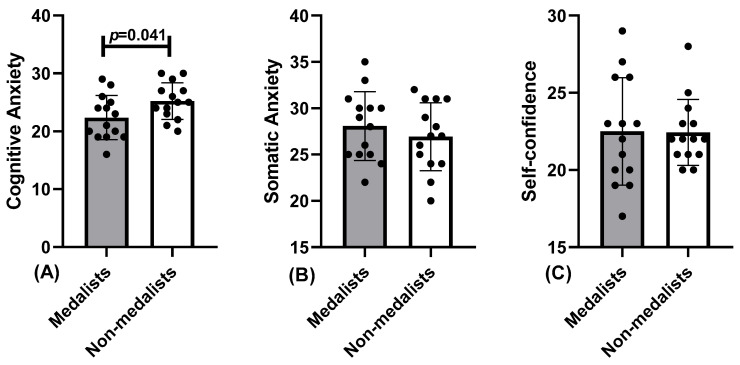
Graphical representation of (**A**) cognitive anxiety, (**B**) somatic anxiety, and (**C**) self-confidence differences between medalist and non-medalist BJJ athletes resulting from the CSAI-2 questionnaire application.

Figure 2 depicts the pre-competitive anxiety results from the SCAI-2 questionnaire. Significant differences (*p* < 0.05, Cohen’s “d” = 0.95, moderate effect) were found only in cognitive anxiety between medalists (22.36 ± 3.82) and non-medalists (25.21 ± 3.17). Cognitive anxiety and somatic anxiety were within normal limits. However, self-confidence did not meet the criteria for normality.

**Figure 3:**
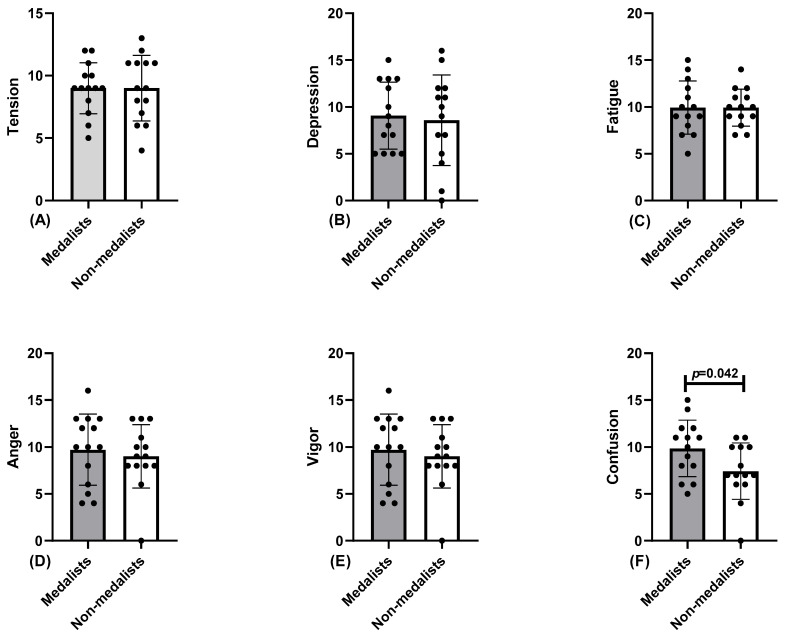
Graphical representation of mood states differences between medalist and non-medalist BJJ athletes resulting from the BRUMS questionnaire. (**A**) tension, (**B**) depression, (**C**) fatigue, (**D**) anger, (**E**) vigor, (**F**) confusion.

Figure 3 shows the mood state results from the BRUMS questionnaire. Significant differences (*p* < 0.05, Cohen’s “d” = 0.81, moderate effect) were found only in mental confusion between medalists (9.86 ± 3.01) and non-medalists (7.43 ± 3.01). Tension, Depression, Fatigue and Confusion, were within normal limits. However, Anger and Vigor did not meet the criteria for normality.

## 4. Discussion

This study aimed to compare pre-competitive anxiety levels and mood states between medalist and non-medalist BJJ athletes graded as white belts. Our primary findings were that non-medalist athletes presented higher levels of pre-competitive anxiety than medalist levels; and medalist athletes reported higher mental confusion before the fight than non-medalist athletes.

CSAI-2 results indicate that non-medalist athletes suffered more cognitive anxiety before the fight than medalist athletes. Cognitive anxiety has been demonstrated to have a negative correlation with sports performance [20]; however, some results in combat sports research are confounding. Contrary to our findings, Papacosta et al. [21] found that medalists judo athletes presented higher cognitive anxiety than non-medalists before an international competition fight. On the other hand, the results of Alejo et al. [7] are partially in line with ours. They found that in juvenile boxing athletes, cognitive anxiety was higher in non-medalists than medalists before a national stage fight. However, for adult athletes, non-medalists presented lower cognitive anxiety than medalists. This variety of results can be related to the influence of other factors on cognitive anxiety and sport performance. A study from Fernández et al. [22] suggests that the expertise level of a fighter could be negatively related to cognitive anxiety. In this line, Faro et al. [23] compared pre-competitive anxiety in BJJ athletes by their graduation level, including blue, purple, brown, and black belts. Interestingly, they do not find statistical differences in any anxiety dimension. This result cannot support the relationship between cognitive anxiety and expertise; however, it is expected that white-belt BJJ athletes have low expertise, and Faro et al. did not address this population. Then, it is possible that higher cognitive anxiety in non-medalists was related to a lower expertise level in the BJJ practice than their medalist counterparts, which led to lower sport performance and the achieved negative result. In addition, both of our groups presented average (medium) cognitive anxiety levels, which is in line with Ghorbanzadeh & Bayar [24] results. They found average cognitive and somatic anxiety levels in male and female taekwondo fighters 1 h before a competition. These results are expected since anxiety is a common emotional response during competitions [11].

Results from the BRUMS questionnaire indicate that medalist athletes presented higher mental confusion than non-medalist athletes. Mental confusion is considered a negative mood dimension that impairs the ability to regulate attention and emotions, and is characterized by disorientation and uncertainty [14]. Studies on wrestlers, marathon runners, and oarsmen have shown that elite athletes exhibit lower levels of confusion than non-athletes [25]. As our study included beginner athletes, it is not strange that this would be different. However, Costa et al. [26] compared pre-competitive mood states in young beach volleyball players, and, contrary to our results, they found that non-medalist athletes presented higher confusion than medalist. In addition, they identified mental confusion as the main indicator of psychological performance. This makes us think that the confusion levels in our athletes would be related to other factors, like the emotional context of the competition. In this line, Hassmén & Blomstrand [27] measured the mood state of football players during a competitive season and, regardless of match outcome, athletes reported greater confusion before the first game of the season compared to later games. In our case, the measured competition was probably one of the first national championships of our white-belt BJJ athletes. Then, a certain level of confusion would be expected even in medalist athletes.

Taken together, our findings show the importance of assessing psychological factors during a competition in less-experienced athletes. Given the relationship between anxiety and performance, it was expected that non-medalist athletes would have higher anxiety levels; however, medalists presenting higher mental confusion was a non-expected result. Further research on this topic is needed to elucidate whether mental confusion could be a common response in less-experienced athletes who are participating in their first national competitions. Finally, a limitation of our study is the lack of specific data from our sample, such as age or BJJ practice experience. This data could help to execute further analyses like comparisons in anxiety and mood states by age or time of expertise in sport.

Our study has some limitations, among which we highlight the small sample size, since we only included weight categories for which we were able to collect data from medalists and non-medalists. Furthermore, our study was limited to collecting data before the competition, making it impossible to follow the athletes during and after the fights. Another limitation relates to the lack of control over prior experience in competitions; perceived importance of the competition; and competitive expectations. And lastly, we did not control for confounding variables such as age; weight category; training experience; number of fights; sequence of fight results; and opponent skill level.

## 5. Conclusions

Pre-competitive anxiety and negative mood states can affect performance during a competition. White-belt BJJ medalists presented less pre-competitive anxiety and higher mental confusion than non-medalists before a fight in a national competition. As the relationship between pre-competitive anxiety and sport performance has been proven, confounding results were found about mental confusion in medalist athletes. Further research is needed to elucidate whether this was a particular phenomenon or mental confusion can be common in less-experienced medalists. However, due to the small sample size, limited control of confounding variables, and exploratory design, these results should be interpreted with caution.

## Figures and Tables

**Table 1:** Sample characterization.

	Medalists	Non-Medalists.	*p*	ICC	CV	α
Age (years)	20.33 ± 1.50	20.31 ± 1.93	0.317	0.177	0.085	0.331
Body mass (Kg)	73.23 ± 6.89	70.89 ± 6.88	0.886	0.053	0.093	0.421
Experience (years)	1.06 ± 0.12	1.02 ± 0.70	0.135	0.009	0.400	0.016

*p* < 0.05 (independent “*t*” test). ICC: Intraclass Correlation Coefficient, CV: Coefficient of Variation.

## Data Availability

The data that support this study can be obtained from the address: www.ufs.br/, Department of Physical Education, accessed on 5 March 2026.
